# The Effect of Conflict on Medical Services for People Living With Advanced HIV Disease and Cryptococcal Meningitis in Ethiopia's Tigray Region: A Qualitative Study of Healthcare Providers

**DOI:** 10.1093/ofid/ofag058

**Published:** 2026-02-13

**Authors:** Temesgen Nurye, Mengistu Hagazi, Gerome B Vallejos, Carla Y Kim, Sennay Gebremariam, Abraha Gebreegziabher, Kibrom Gebreselasie, Kathryn B Holroyd, Kiran T Thakur

**Affiliations:** Department of Neurology, Addis Ababa University, Addis Ababa, Ethiopia; School of Public Health, Mekelle University College of Health Sciences, Mekelle, Ethiopia; Program in Neuroinfectious Diseases, Department of Neurology, Columbia University Irving Medical Center, NewYork Presbyterian Hospital, New York, New York, USA; Program in Neuroinfectious Diseases, Department of Neurology, Columbia University Irving Medical Center, NewYork Presbyterian Hospital, New York, New York, USA; School of Public Health, Mekelle University College of Health Sciences, Mekelle, Ethiopia; School of Public Health, Mekelle University College of Health Sciences, Mekelle, Ethiopia; School of Public Health, Mekelle University College of Health Sciences, Mekelle, Ethiopia; Program in Neuroinfectious Diseases, Department of Neurology, Columbia University Irving Medical Center, NewYork Presbyterian Hospital, New York, New York, USA; Program in Neuroinfectious Diseases, Department of Neurology, Columbia University Irving Medical Center, NewYork Presbyterian Hospital, New York, New York, USA

**Keywords:** advanced HIV disease, armed conflict, cryptococcal meningitis, healthcare providers, health system disruption, qualitative research

## Abstract

**Background:**

Armed conflict in Tigray, Ethiopia, disrupted healthcare, and heightened risks for people with advanced HIV disease (AHD) including serious infections like cryptococcal meningitis (CM). This study described healthcare providers' experiences before, during, and after the conflict, focusing on vulnerabilities in HIV and CM services, infrastructure, and supply availability.

**Methods:**

This qualitative study was conducted at Ayder Comprehensive Specialized Hospital in Tigray, Ethiopia. Healthcare professionals were purposively sampled across outpatient and inpatient settings. Semistructured interviews explored care delivery, resource challenges, and professional experiences across preconflict, conflict, and postconflict phases. Transcripts underwent inductive thematic analysis, with iterative coding and team consensus to identify key themes reflecting participants' experiences.

**Results:**

Interviews with 13 healthcare professionals highlighted 4 main findings: (1) the conflict disrupted HIV and CM services, causing drug shortages, treatment interruptions, and preventable suffering; (2) providers faced significant emotional and moral distress while delivering care in conflict settings; (3) essential medications, diagnostic tools, and standardized supplies for CM care were largely unavailable at all phases of conflict; and (4) gaps in training and limited adherence to CM guidelines hindered effective patient management.

**Conclusions:**

This study described frontline providers' experiences of HIV and CM care before, during, and after the northern Ethiopian conflict. Findings revealed disrupted services, severe shortages of essential medications and diagnostics, provider moral distress, and limited adherence to CM guidelines. Highlighting the urgent need to rebuild healthcare infrastructure, secure uninterrupted HIV and CM treatment, strengthen evidence-based training, and provide sustained psychosocial and professional support for healthcare providers.

## Teaser

This qualitative study in Tigray, Ethiopia, explores healthcare providers' experiences delivering advanced HIV and cryptococcal meningitis care before, during, and after conflict, revealing disrupted services, drug shortages, and emotional distress, highlighting urgent needs for resilient systems, uninterrupted care, and provider support.

Advanced HIV disease (AHD), defined by WHO as a CD4 count <200 cells/mm^3^, is associated with a markedly increased risk of opportunistic infections (OI) [1]. Among these, cryptococcal meningitis (CM) is a leading cause of death, responsible for nearly 625 000 deaths each year, 73% of which occur in sub-Saharan Africa [2]. Timely diagnosis and treatment are critical for the effective management of CM. In settings affected by armed conflict, numerous studies show the impact of the dismantling of healthcare infrastructure, leading to reduced access to essential medications, loss of trained healthcare personnel, and breakdowns in supply chains [3–5]. In South Sudan, HIV cases have surged to 48 000 and only 34% of healthcare facilities providing HIV services remain functional. Similarly, in Syria, there has been a 50% decline in HIV/AIDS surveillance activities, including voluntary counseling and testing services [6–8].

In Tigray, HIV prevalence was projected at 1.4% before the outbreak of the armed conflict [9]. Since the armed conflict began in November 2020, HIV services have been disrupted, and access to medical supplies has been restricted, increasing the burden of AHD across northern Ethiopia. Antiretroviral therapy (ART) drug supplies dropped by 68.8%, while laboratory testing fell dramatically from nearly 10 000 tests in 2018–2019 to 3780 in 2021, and none in 2022 [10]. Furthermore, a recent report documents the region's fragile healthcare system, leaving thousands of people with HIV without access to essential care [11].

This study aimed to describe the experiences of frontline health care providers before, during, and after the conflict, focusing on vulnerabilities in HIV and CM services, healthcare infrastructure, and supply reserves.

## METHODS

### Study Design

This study was conducted at Ayder Hospital, a tertiary referral center with a catchment area of 9 million people in the Tigray region of Northern Ethiopia. This was a qualitative descriptive study, with a retrospective longitudinal focus, structured into 3 distinct temporal phases: preconflict, during conflict, and postconflict.

### Study Population

This study included a diverse group of healthcare professionals directly involved in the care of HIV patients, including those with CM across both outpatient and inpatient settings. Participants were purposely selected based on their active role in the diagnosis, treatment, medication dispensing, laboratory investigation, and overall management of AHD. All participants who were involved in the care of AHD were approached and those who were willing to give the interview were selected as participants. Active enrollment was conducted through departmental emails and communication with departmental heads and staff by authors affiliated with Ayder Hospital.

To ensure a wide range of perspectives, participants were recruited from various professional backgrounds, including neurologists, internists, internal medicine residents, clinical nurses, and pharmacists working in HIV care units. In the inpatient setting, department heads helped identify key informants with substantial clinical experience. In the outpatient ART clinic, providers were approached directly based on their availability and engagement in routine HIV care.

### Data Collection

A semistructured interview guide was developed to facilitate in-depth exploration of CM care within the broader context of AHD. The guide was informed by a review of existing literature, expert consultation in HIV and neuroinfectious diseases, and input from implementation scientists with expertise in qualitative methods and health systems research. To avoid the constraints of a questionnaire or survey, we included open-ended questions designed to explore perceptions of CM care before, during, and after the conflict, as well as changes in clinical practice, resource availability, care delivery challenges, emotional and professional responses, and team-based coping strategies. Though not a standardized questionnaire, this semistructure interview guide underwent numerous revisions to maintain flexibility and encourage response-guided tangents, allowing the addition of follow-up questions as new themes emerged during the interviews.

Interviews took place in November 2024 to February 2025, ∼2 years postconflict. The interviews took up to 2 hours and were conducted in person by author TN, a neurologist in clinical research fellowship training through Columbia University Medical Center. With participant permission, interviews were audio-recorded to ensure accuracy. All interviews were conducted in Amharic or Tigrigna, the local languages spoken by participants, to encourage open, comfortable dialogue. Interviews were conducted in quiet, private settings to maintain confidentiality and encourage candid discussion.

Interviews were then translated into English for analysis. Translation was carried out by 2 researchers and 1 clinician who are fluent in both Amharic and English. All 3 had prior experience translating qualitative health research and were actively involved in HIV care, ensuring that technical and contextual meanings were preserved. To ensure consistency and accuracy, the translated transcripts were reviewed line by line by a 4th team member with qualitative research training and bilingual fluency.

### Data Analysis

We conducted an inductive thematic analysis. Study members read all transcripts to ensure deep familiarization with the content, after which open coding was used to identify and label quotable units of texts across transcripts. Open coding was conducted independently by 2 coders to compare and consolidate codes following discussion. Codes were iteratively reviewed and grouped into broader categories and themes that reflected participants' experiences in preconflict, conflict, and postconflict periods. Thematic development followed an inductive approach, guided by patterns emerging from the data rather than any predefined theoretical framework. Code and thematic saturation was assessed through continuous analysis and determined when no new codes or themes were identified from consecutive interviews, Saturation was achieved by the 13th interview. These categories were further refined into overarching themes through a series of team meetings involving 4 research team members. Discrepancies in coding and theme categorization were discussed until consensus was reached, enhancing both the credibility and reflexivity of the analysis. Trained research assistants conducted transcription, and all transcripts were cross-checked against audio recordings by at least one team member for accuracy.

### Ethical Approval and Patient Consent Statement

Ethical approval [MU-IRB 2445/2025] for this qualitative study was obtained from the Institutional Review Board (IRB) of Ayder Hospital, affiliated with Mekelle University College of Health Sciences. The IRB reviewed the study objectives, interview guides, and informed consent process to ensure alignment with ethical standards for research involving human participants. All participants were provided with detailed information about the purpose of the study, their voluntary participation, the confidentiality of their responses, and their right to withdraw at any time without any consequences. Written informed consent was obtained before each interview. To protect participants' identities, all transcripts were anonymized and stored securely with access limited to the core research team. Study participants were not compensated for participation.

## RESULTS

A total of 13 healthcare professionals were interviewed, comprising of senior clinicians, internal medicine residents, nurses, and pharmacists. Participants' median age was 33. The majority were male (10/13), with 3 female participants (Table 1).

**Table 1.** Sociodemographic Characteristics of Study Participants in Advanced HIV Care in Ayder Comprehensive Specialized Hospital

Characteristics		Number (Percent)
Age in years (median, interquartile range)	…	33 (9.5)
Sex	Male	9 (77%)
	Female	3 (23%)
Educational status	MSc	2 (15%)
	BSc	3 (23%)
	Specialist/subspecialist	8 (62%)
Work experience in years (median, interquartile range)	Median (interquartile range)	8 (15)
Department	Nursing	3 (23%)
	Pharmacy	2 (15%)
	Internal Medicine	8 (62%)

Educational levels varied from bachelor's to master's degrees and sub-specialty training. Participants' clinical experience spanned from 3 to 30 years. They represented several departments, including internal medicine, neurology, adult nursing/ART outpatient services, medical wards, and pharmacy.

Four key themes emerged: (1) Impact of armed conflict on HIV/CM Care; (2) Impact of armed conflict on health workers; (3) Availability of resources for HIV care; (4) Training gaps and implementation of guidelines (Table 2).

**Table 2.** Selected Themes and Illustrative Quotes From Qualitative Interviews

Health Professional	Topic	Quote	Theme/Insight	Context
Nurse	Impact of Armed Conflict on HIV Care	“Some patients were newly diagnosed, but we couldn’t start them on HAART due to a shortage of medications”	Serious shortage of laboratory services such as CD4, Viral load, and ARV drugs during the conflict	The armed conflict in disrupted the HIV care
Senior clinician		“Many patients were not able to continue their HIV drugs, and some were even taking whatever was available. Patients were taking drugs whose expiry date had passed”		
Resident Physician	Postconflict Improvements and Remaining Gaps	“CD4 counts are available, but there are consistency problems. The CrAg test is now recently accessible. In terms of treatment, only fluconazole is available, and we’ve been told that amphotericin B will be available soon”	CD4 testing and viral load are inconsistently available	Improvement is seen in service provision, with ongoing challenges in HIV/CM care
Resident physician	Challenges in CM Diagnosis and Treatment	“We still don’t have an LP set, and we use IV cannulas to perform LP, and the results we get from using IV cannulas are unreliable”	Ongoing challenge in accessing LP set, Indian ink, CrAg test, and antifungal medications	CM prevention, diagnosis, and treatment have been a continuous challenge at Ayder Hospital before, during, and after the conflict
Senior clinician		“We don’t even have the proper India ink staining, and we were even using a dye not meant for the procedure as an India ink”		
		“Fluconazole is available. Ambisome is still not available, and flucytosine, I have never seen it being given to patients. I have not seen the drug itself, I know it in books”		
Nurse	Training Gaps	“I’ve had orientation training once on CM, which was not enough, and I had to update my colleagues back at my hospital”	The absence of training of health professionals about the prevention and diagnosis of CM compromises the care	Capacity building and professional development of health professionals in the care of HIV, particularly CM, at Ayder Hospital is lacking
Nurse		“Regarding CM before the conflict, we had little knowledge of CM management. Even now, we still have very little understanding. We don’t even know, for example, if prophylaxis is needed. We only recently began testing for CrAg in the past 2 months. If the CrAg turns positive, we just advise them on ARV drug adherence. CM is a fatal condition, second only to TB meningitis, but we still have very little understanding of it		
Resident physician		“We often don’t trust the lab results because of the reliability issues, so we use our clinical judgment to start treatment”		
Pharmacist	Psychological Impact of the Armed Conflict on Healthcare Workers	“After we would go home, we couldn’t stop thinking about it. We are all still traumatized”	Psychological Trauma in health Professionals in the Aftermath of armed conflict	The conflict negatively affected the emotional well-being of the health professionals

### Theme 1: Impact of Armed Conflict on HIV/CM Care

Hospitals experienced a critical shortage of antiretroviral medications and antifungal medications needed to treat CM. The disruption of healthcare infrastructure, transportation, and pervasive insecurity severely impacted the continuity of care. As a result, many patients suffered preventable complications.

A senior clinician recalled that period:

“…During the armed conflict, when we were unable to do anything, we lost young patients to cryptococcal meningitis and other opportunistic infections…” [IDI11]

A medical resident echoed:

“…Many HIV patients died upon arrival at the hospital. There was no transportation, no medication, and no safety. We were powerless to help…” [Medical Resident, IDI7]

A nurse recalled the condition:

“Some patients were newly diagnosed, but we couldn’t start them on HAART due to a shortage of medications.”

Another clinician added:

“Many patients were not able to continue their HIV drugs, and some were even taking whatever was available. Patients were taking drugs whose expiry date had passed”.

### Theme 2: Impact of Armed Conflict on Healthcare Workers

Healthcare workers endured profound physical, emotional, and psychological suffering during the conflict, facing the challenge of acting both as caregivers and victims of conflict. As members of the affected community, they lived through insecurity. As professionals, they were forced to navigate an impacted healthcare system. The disruption of infrastructure, and shortages of life-saving medications and supplies left healthcare providers overwhelmed. Many experienced lasting moral distress from witnessing the preventable suffering of their patients.

A pharmacist described the situation:

“…During the conflict, health professionals faced a dire shortage of medical supplies. We tried to help, working both office hours and duty shifts, but without resources, we were powerless. Watching patients cry, and suffer, while knowing what they needed but being unable to provide it was heartbreaking. It left us hopeless…” [Pharmacist, IDI12]

A nonphysician clinician added:

“…It's difficult to describe how heartbreaking the situation was. We felt helpless. It was devastating to have nothing to offer when patients needed us the most. Personally, it was an emotional and painful period. Professionally, it drained us all.” [Non-physician clinician, IDI5]

A medical resident highlighted the broader personal and professional consequences:

“…The conflict affected me and many others in so many ways. I couldn’t do what I was trained to do, and I didn’t receive what I was owed. We’ve been wounded morally, emotionally, and economically…” [Medical Resident, IDI6]

### Theme 3: Availability of Resources

Before the outbreak of the armed conflict, essential resources for HIV care, including CD4 and viral load testing, as well as ART and medications for OIs, were largely available and functional. Participants consistently described the preconflict period as one of relative stability and efficiency in service delivery.

One nonphysician clinician recounted:

“Before the armed conflict, HIV care was well organized. We had a CD4 machine in the ART clinic laboratory, we were able to get viral load results on time, and we had a steady supply of medications, not just for ART but also for opportunistic infections. We were all very happy with the work we were doing, and the patients were satisfied with our services. There was no shortage of resources.” Non-physician clinician, IDI P1

A pharmacist working in the pharmaceutical supply chain confirmed this:

“Before the conflict, all necessary HIV drugs and other resources that can help in overall HIV care were available. Investigations for HIV, including CD4, were functional.” Pharmacist, IDI11

Another participant added:

“Before the conflict erupted, almost all services were provided. ART was fully functional and had never been interrupted in Ethiopia, including Tigray. Laboratory investigations like CD4 and viral load were functional most of the time,

One clinician recalled the shortage of resources during the conflict:

“During the conflict, we couldn't perform organ function tests before starting treatment, and we weren’t concerned about the side effects of the drugs. Some patients were newly diagnosed, but we couldn’t start them on [Highly Active Antiretroviral Therapy] HAART due to a shortage of medications. We were only able to distribute medications for short periods, sometimes only for a week or a few days.” Non-physician clinician, IDI P1

Senior clinician

“Many people were displaced internally and sought refuge in places like Mekelle, which put additional pressure on our already limited resources.” senior clinician, IDI2

Regarding CM, the availability of essential medications was a persistent issue across all 3 periods before, during, and after the conflict. With more consistent availability than Flucytosine and liposomal Amphotericin B before the conflict, fluconazole was the standard antifungal treatment for CM cases. While Amphotericin B and Flucytosine are reportedly available in the pharmacy inventory after the conflict, frontline physicians stated that there are inconsistencies in the availability of these drugs in practice. Consequently, treatment of CM relies almost entirely on Fluconazole, which itself was inconsistently available during the conflict.

“Before, during, and even after the armed conflict, we have never seen the first-line drugs for CM (Amphotericin B and Flucytosine). We were using Fluconazole to treat CM before the conflict, during, and even now. But during the conflict, even Fluconazole was difficult to get.” Internist, IDI11

A senior clinician said:

“Fluconazole is available. Ambisome is still not available, and flucytosine, I have never seen it being given to patients. I have not seen the drug itself, I know it in books.”

As far as diagnostic tests for CM, there have been some improvements in diagnostic availability postconflict, particularly with serum and cerebrospinal fluid cryptococcal antigen (CrAg) tests, access remains inconsistent:

A senior clinician described the condition as follows:

“We don’t even have the proper India ink staining, and we were even using a dye not meant for the procedure as an India ink.”

Another major gap highlighted was the absence of standard lumbar puncture (LP) kits throughout all periods of time. Providers reported using improvised tools such as intravenous (IV) cannulas, which significantly increased the failure rate of the procedure and compromised diagnostic accuracy for CM.

A medical resident expressed frustration:

“We don’t have a standard set, but we do it with an IV cannula or syringe, and the failure rate is high, maybe as much as 50%.”Medical Resident, IDI7

### Theme 4: Training Gaps and Guideline Adherence

Most participants noted that before the outbreak of the conflict, there were some opportunities for training, particularly in outpatient HIV care. These included locally organized in-service trainings, occasional national workshops, and limited international mentorship opportunities provided through partnerships with nongovernmental organizations (NGOs) such as the International Center for AIDS Care and Treatment Program (ICAP) at Columbia University. However, participants emphasized that these initiatives were infrequent, narrowly focused, and largely excluded topics like CM. Following the onset of the conflict, nearly all such opportunities disappeared due to insecurity, disruption of institutional functions, and the withdrawal of external support.

A participant from the HIV outpatient clinic reflected on the situation before the conflict:

“Before the conflict, there were a few training sessions that focused mainly on HIV/TB and pediatric HIV care. Since then, there has been no additional training.” Non-physician clinician, IDI P1

Many interviewees reported that they had never received any formal training on CM at all periods. Instead, they relied on self-directed learning through books and articles. A consistent across the preconflict, conflict, and postconflict periods.

A nurse from the ART outpatient department noted:

“There was a one-day training session before the conflict that one of my colleagues attended. He then shared what he learned with us, such as the importance of testing for CrAg.” Non-physician clinician, IDI P1

A clinician working in both the infectious diseases outpatient clinic and inpatient wards expressed concern about the general level of knowledge among providers:

“Many professionals do not have enough knowledge about CM care and investigation. They practice without adhering to the current updated guidelines for the diagnosis and management of CM. Some providers update themselves, which may not be sufficient.” IDI13

Difficulties with CM guideline adherence are further exemplified by the experience of a senior nurse working in the inpatient ward:

“I am hearing about the CM guideline for the first time from you. We do not have any information about the guidelines for cryptococcal meningitis.” Nurse, IDI10

The implementation of the updated CM guidelines at Ayder Hospital remains suboptimal. Most participants reported that the current guidelines are not actively disseminated or systematically followed in clinical practice. Instead, clinical decision-making is often based on personal initiative, such as informal reading, rather than structured institutional guidance.

## DISCUSSION

In this work, we explore the profound and multifaceted impact of armed conflict on the delivery of care for HIV and CM in a tertiary healthcare facility in northern Ethiopia. Through the experiences of frontline healthcare providers, the findings reveal how systemic disruption, resource scarcity, and psychological burden intersect to compromise both patient outcomes and health worker well-being.

Our study adds to the growing body of evidence demonstrating the profound effects of armed conflict on healthcare systems. For example, another study from the Tigray conflict showed that poor HIV treatment outcomes—such as treatment failure, lost to follow-up, and death—nearly doubled during the conflict, coinciding with a drop in ART availability and sharp declines in CD4 and viral load testing [10].

Although there have been no dedicated studies on CM in the context of conflict, diagnosis and treatment are closely tied to the broader HIV care infrastructure. As such, disruptions to HIV services likely have direct implications for CM outcomes. In our work, providers reported severe shortages of ART, near-total absence of amphotericin B and flucytosine, lack of diagnostic tools, and first-line antifungals. CT and MRI machines were nonfunctional during the conflict, and the absence of LP kits forced providers to rely on improvised tools with high failure rates. These findings echo broader evidence from other conflict-affected regions, where destruction of institutional infrastructure frequently interrupt access to life-saving medications and diagnostics [10, 12].

Another key finding was the persistent gap in formal training and preparedness for CM. Despite the availability of updated CM guidelines, implementation was limited. Most participants were unaware of formal protocols, and clinical care was based on informal practices, self-directed learning and informal peer exchanges rather than standardized guidance. This absence of structured education and supervision contributed to inconsistent clinical practice. These challenges mirror findings from other conflict-affected settings, where insecurity, workforce displacement, and institutional collapse disrupt medical training and knowledge transfer [13, 14].

The conflict also had a devastating impact on HIV patients, creating cascading barriers to timely care. Displacement of patients and lack of transportation made it difficult for patients to access care, often delaying presentation until they were critically ill. Providers recalled how patients arrived at healthcare facilities already in advanced stages of disease, some with CM only to succumb to preventable complications. Stigma and fear, exacerbated by the instability and discouraged care-seeking behavior. These disruptions extended beyond individual outcomes and likely contributed to increased HIV transmission and a surge at a population-based level in OIs in the region [15]. These findings are consistent with trends seen in other conflict settings, where ongoing instability has contributed to increased HIV transmission [16, 17].

Perhaps one of the most under-recognized but critical insights from this study is the profound psychological impact the conflict had on frontline health professionals. Providers faced moral injury knowing what care was needed but simultaneously being unable to deliver it, as well as trauma from witnessing preventable sufferings. Many also endured personal hardship, including psychological distress. This dual burden, both professional and personal, has long-term implications for workforce retention, mental health, and postcrisis health system rebuilding. Greater investment in psychosocial support, financial security, and professional recognition for health workers in conflict settings is essential to sustain their resilience and recovery [18–20].

Postconflict recovery is often unevenly distributed, with health systems struggling to reestablish dependable supply chains. In many settings, persistent stockouts, procurement delays, and poor logistical coordination continue to disrupt access to essential services, including HIV and CM care. These challenges have been further exacerbated by the recent freeze on President's Emergency Plan for AIDS Relief funding in early 2025, which has raised urgent concerns about the continuity of HIV services across sub-Saharan Africa. A modeling study involving 7 high-burden countries, including Ethiopia, projected that sustained funding disruptions could result in approximately 74 000 additional HIV-related deaths and more than 100 000 new infections by 2030 [21]. Even brief interruptions in support can further destabilize already fragile systems, making it significantly harder to manage AHD and life-threatening OIs like CM.

Before the armed conflict, Ayder Hospital relied on established local and national supply chains and distributors to stock their diagnostic laboratories and pharmacies that were supplemented by donations of LP kits, and CD4 testing equipment from international NGOs. With supply lines cut during the conflict, strategies to strengthen resilience in crises require more than restoring preconflict functionality; it demands structural reforms such as flexible procurement policies, decentralized storage, and regional buffer stocks to cushion against future disruptions. Countries like Nigeria, eSwatini, and Zambia have successfully implemented decentralized drug distribution, improving HIV service continuity by bringing medicines closer to patients, reducing treatment interruptions, and decongesting health facilities [22–24]. Similarly, in Haiti community-based HIV care models have been showed to function effectively despite prolonged periods of political instability and civil unrest [25]. These successes suggest that broader adoption of decentralized drug distribution could help maintain care continuity during crises, ensuring uninterrupted access to essential medications. Hence, sustained donor engagement, predictable funding streams, and the integration of emergency preparedness into national health plans are critical to long-term stability.

Our study offers important insights into HIV and CM care in a conflict-affected setting; however, several limitations should be acknowledged. First, the study was conducted at a single tertiary hospital in northern Ethiopia, which may limit the transferability of findings to other regions or healthcare systems with different resource levels or conflict dynamics and may not reflect the realities of rural or lower-level health facilities where service disruptions could be even more pronounced. Second, the interviews were conducted nearly 3 years after the conflict, which introduces a significant risk of recall bias. Participants were asked to reflect on experiences across 3 distinct periods, memory challenges, emotional distress, or erosion of detail may have led to inaccuracies or incomplete accounts. Third, although we included a range of healthcare professionals, the perspectives of patients and caregivers were not explored, limiting our understanding of lived experiences from the user side of the health system. Fourth, social desirability bias may have influenced responses, especially in discussing institutional practices or gaps in clinical knowledge. Despite efforts to create a safe and confidential interview environment, some participants may have been hesitant to disclose sensitive information fully.

In summary, the conflict in Tigray exposed deep structural weaknesses in HIV and CM care and the immense strain on the health workforce. Moving forward, recovery efforts must focus on restoring infrastructure, embedding evidence-based protocols, maintaining continuous care for patients with HIV, and providing sustained psychosocial support for healthcare providers. These findings offer valuable lessons not only for Ethiopia but for all health systems navigating the challenges of conflict and postconflict recovery.
